# Alimentary Tract Atresias associated with Anorectal Malformations: 10 Years' Experience

**DOI:** 10.21699/jns.v5i4.449

**Published:** 2016-10-10

**Authors:** Manoj Saha

**Affiliations:** Department of Pediatric Surgery, Gauhati Medical College, GUWAHATI, ASSAM, INDIA

**Keywords:** Anorectal Malformation, Esophageal atresia, Duodenal atresia, Associated anomalies

## Abstract

Anorectal malformation (ARM) is one of the most common congenital anomaly that requires emergency surgery in the neonatal period. ARMs are frequently associated with other life threatening congenital anomalies. Commonly associated anomalies are genito-urinary, cardiovascular, gastro-intestinal, skeletal and spinal. Alimentary tract anomalies are frequently masked by the intestinal obstruction produced by the anorectal atresia. This retrospective study was carried out to find out the incidence of associated alimentary tract atresias with ARM. In our series, out of 785 cases of high ARM, 14 cases had associated esophageal atresia (1.8%), followed by 7 cases of duodenal atresia (0.89%), and followed by pyloric atresia, jejuno-ileal atresia and colonic atresia.

## INTRODUCTION

Anorectal malformation is frequently associated other congenital anomalies; amongst which urological anomalies are the most common. Most of the associated anomalies are not life threatening and do not need priority treatment over anorectal malformation. But Alimentary tract atresias are life threatening and are frequently overshadowed by the more distal anorectal atresias which are quite obvious. We encountered 26 cases of alimentary tract atresias associated with high anorectal malformation during last 10 years. This retrospective study was carried out to find out the incidence of associated alimentary tract atresias, how they were diagnosed and treated in a limited resource pediatric surgical facility.

## MATERIALS AND METHODS

From January 2006 to December 2015 all cases of anorectal malformations treated primarily in our institute were analyzed. Cases associated alimentary tract atresias were picked up for detail analysis according to gender, type of anomalies (high, intermediate, or low), type of associated alimentary atresia, timing of their diagnosis [Pre, per and post operative], treatment done and their outcome. As the total number of associated alimentary tract atresia was very less and varied; individual types are described in brief in the result section. 

## RESULTS

From January 2006 to December 2015, 950 cases of anorectal malformations were treated. There were 785 cases of high and intermediate cases and 165 low cases. Low cases were not associated with alimentary tract atresias in our series. High cases were associated with various alimentary tract and non- alimentary tract anomalies. Total 26 cases of intermediate and high ARM cases had associated alimentary tract atresias. Amongst alimentary tract atresias esophageal atresia was the single most common type (n=14), followed by duodenal atresia (n=7), pyloric atresia (n=2), jejuno-ileal atresia (n=2) and colonic atresia (n=1). Salient features of individual type of associated alimentary tract atresias are described below. Different associated atresias are shown in table 1.

**Figure:**
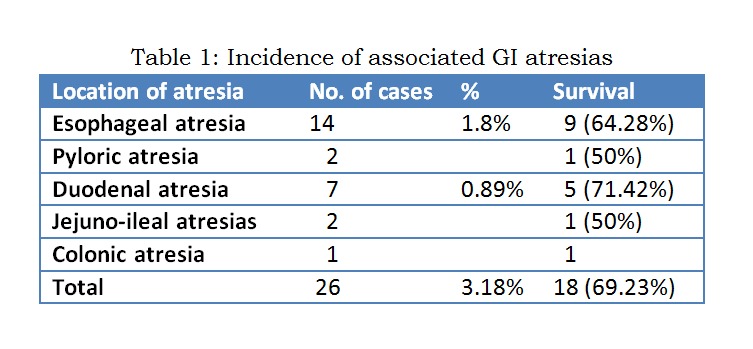
Table 1: Incidence of associated GI atresias

Esophageal atresia: There were fourteen cases of esophageal atresia with tracheo-esophageal fistula (EA+TEF). There were 13 male and 1 female patients. Eleven cases were referred to us as anorectal malformation only. Two male babies and the one female baby were referred as associated esophageal atresia also. Esophageal atresia was suspected due to excessive salivation and were diagnosed by failure to pass an esophageal tube and confirmed by radiography. All the cases of esophageal atresia were of Type-C, 12 of the cases were full term and above 2 kg of weight with moderate pneumonia; 2 male cases were preterm, low birth weight (< 2kg). All 12 full-term male cases were treated by primary repair of the esophagus and colostomy. One female case with recto-vestibular fistula was decompressing well, was treated by primary repair of the esophagus in the neonatal period and PSARP three months later. One preterm baby died before operation, for another preterm baby colostomy was done first, under local anesthesia but the patient died 48 hours after operation due to pneumonia. Three full term baby died in the post operative period, two due to pneumonia and other due to leakage and sepsis. All the cases survived and are doing well during follow up period. Survival rate for associated EA+TEF was 64.28%.

Duodenal atresia: There were seven cases of duodenal atresia. All the cases were diagnosed by pre-operative abdominal skiagram showing typical double-bubble sign (Fig.1). All the cases were male. Two cases had associated Down syndrome. Two cases were preterm, weighing 1.6 kg and 1.8 kg. Duodeno-duodenostomy was done in four cases and duodeno-jejunostomy in two cases, one preterm newborn died before operation and second preterm child died in the post operative period. Salient features of the cases are shown in table 2.

**Figure:**
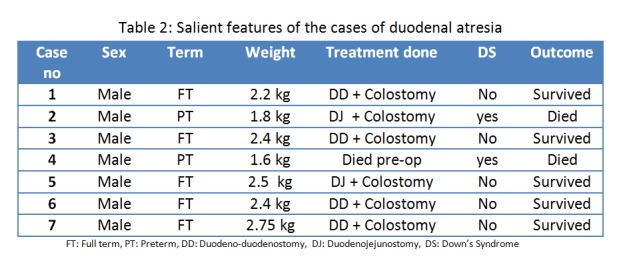
Table 2: Salient features of the cases of duodenal atresia

**Figure:**
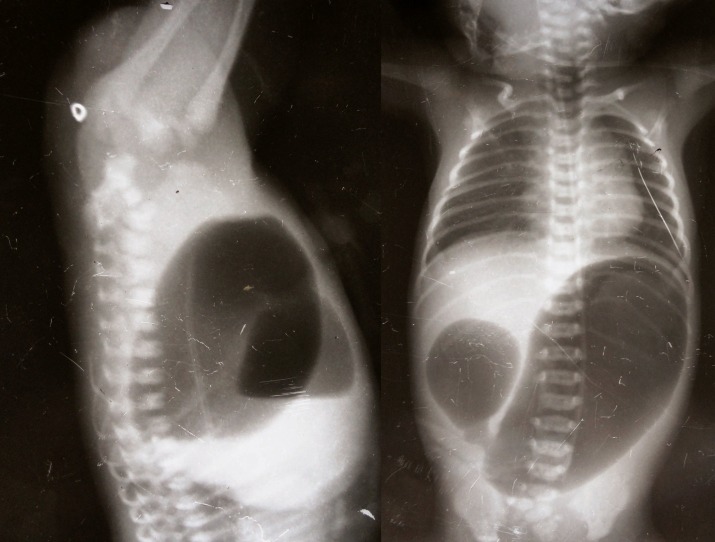
Figure 1: Invertogram showing high ARM and AP view showing duodenal atresia

Pyloric atresia: Two cases had associated pyloric atresia. One male baby was delivered at term; birth weight was 2.5 kg, referred to our facility 48 hours after birth for anorectal malformation. Pre-operative skiagram showed normal stomach gas shadow (nasogastric tube was in situ) and invertogram showed high anorectal anomaly. Accordingly sigmoid colostomy was done. Patient recovered and the colostomy started functioning properly. But the child did not tolerate feed and had non-bilious vomiting. Initially it was thought to be due to postoperative ileus but as vomiting persisted, upper GI contrast radiography was taken and it showed pyloric atresia. We counseled the parents second operation and gastro-duodenostomy was done. Eventually child recovered, tolerated feed and discharged with colostomy. Another male child was preterm, weighing 1.6 kg presented with high ARM with a visible bowel loop in the upper abdomen. We suspected associated bowel atresia. On laparotomy there was pyloric atresia (Fig. 2), nearby duodenum was atretic and therefore gastro-jejunostomy and colostomy was done. Child died in the postoperative period due to respiratory failure. None of our cases had associated epidermolysis bullosa.

**Figure:**
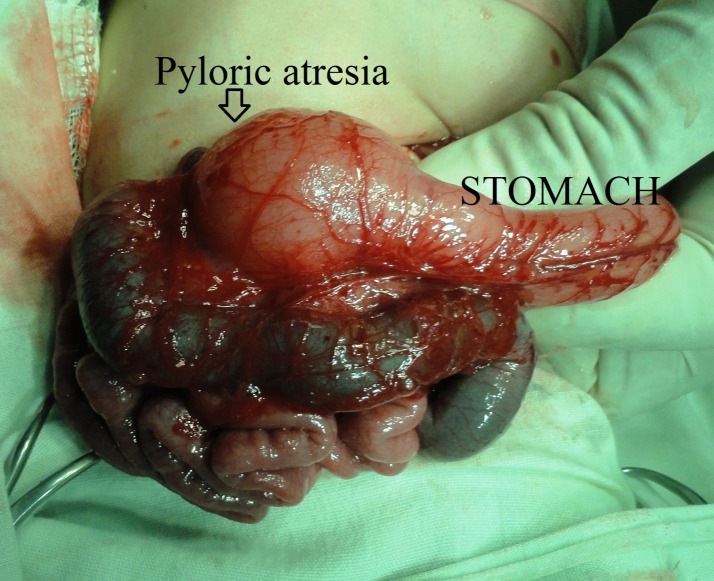
Figure 2: Picture showing pyloric atresia and dilated colon.

Ileal atresia: One male, full-term, 2.8 kg baby had associated ileal atresia. Child had gross abdominal distension at presentation with dilated bowel loops on plain skiagram and therefore, we planned for transverse colostomy. On exploration colon was not found. On further exploration we found distal ileal atresia with 'V' shaped defect in the mesentery and about 10 cm of terminal ileum was present. Double barrel ileostomy was done. Child recovered and discharged. Another preterm, 1.7 kg male baby with high ARM had apple peel type of ileal atresia. During the attempt of colostomy, apple peel type of ileal atresia with precarious blood supply was found associated with microcolon. Bulbous end of the ileum was brought out as ileostomy. Child died in the post operative period.

Colonic atresia: This male child was born at term, presented with anorectal malformation. High sigmoid colostomy was done. Patient recovered and was discharged. During pre-pull through work-up child was found to have distal colonic atresia. Diagnosis was confirmed during laparotomy, distal part of the colon was excised and sigmoid pull through was done through Abdomino-PSARP approach.

## DISCUSSION

Anorectal malformation is one of the most common neonatal surgical emergencies. Many a times these anomalies are associated with other anomalies. Well known associations are VATER, VACTERL and CHARGE. But there is battery of isolated anomalies associated commonly with high and also with low type of anorectal malformations. Externally visible anomalies are quite obvious and are usually not life threatening but internal anomalies like cardiac, alimentary tract and renal anomalies are detected either by routine screening or when they produce significant affect on the patients' systems. Diagnosis of alimentary tract atresias sometimes poses a special diagnostic problem. Esophageal atresia is usually diagnosed in the delivery room either by failure to pass a tube into stomach or by excessive salivation (frothy baby) and regurgitation of all feeds. Out of 785 cases of ARM only 14 (1.8%) cases had associated EA+TEF. But in the literature incidence of associated EA+TEF has been reported as 2.5 to 10% [1, 2]. We managed our 12 cases in one stage by colostomy and repair of EA+TEF. But septic and low birth weight cases are better managed by initial colostomy and repair of EA+TEF when the child more stable [1]. We did initial colostomy in the preterm, low birth weight case, but the child died after colostomy. Overall survival of EA+TEF in our institute is 60%, and in the present study, it was 64.28% when associated with ARM. 

Gastro-intestinal anomalies are associated in 5-10.7% of the cases of ARM [2,3]. Duodenal atresia occurs in 2-3% of the cases of ARM [2]. We encountered 7 cases of duodenal atresia associated with ARM (0.89%). Duodenal atresia is also quite obvious in routine skiagram (double-bubble sign) and by copious gastric aspirate. 

Pyloric atresia is a rare anomaly and to our knowledge, it has not been reported to be associated with ARM. Our first case posed a special problem in diagnosing because of two reasons: 1) non-bilious vomiting and 2) colostomy was functioning properly with passage of bilious stool. In second case we suspected associated bowel atresia but it was not clear preoperatively. 

Associated small bowel atresia is also reported with high and low ARM [2,4]. Intestinal obstruction produced by jejuno-ileal atresias are overshadowed by the obstruction produced by the anorectal atresias. As in our cases, these cases are not diagnoses preoperatively. 

Colonic atresia is very rare as such and when associated anorectal malformation is practically impossible to diagnose preoperatively because of distal location of the atresia. Colonic atresia has been reported as isolated atresia as well as combined small bowel and colonic atresia in association with ARM [2,5-7]. In our case it was not diagnosed in the neonatal period, but revealed after distal loopogram. This speaks for importance of the distal loopogram before definitive pull through.

Overall survival of ARM with associated alimentary tract atresia in our study is 69.23%. We observed that associated alimentary tract atresias did not cause increased mortality if detected and treated in time. Main cause of mortality was associated prematurity and low birth weight. But others have reported significant increased mortality in patients with EA+TEF when they are associated with ARM [1]. This is a small series and incidences of individual associated atresias are also very less and therefore impact of these associated atresias on patients' survival cannot be derived.

## CONCLUSION

We conclude that presence of one obvious obstruction does not exclude the possibility of another obstruction of alimentary tract. Presence of any unusual abdominal sign with ARM should be considered duly and evaluated.

## Footnotes

**Source of Support:** None

**Conflict of Interest:** None
